# An Individual With Hearing Preservation and Bimodal Hearing Using a Cochlear Implant and Hearing Aids Has Perturbed Sound Localization but Preserved Speech Perception

**DOI:** 10.3389/fneur.2019.00637

**Published:** 2019-06-21

**Authors:** Snandan Sharma, Lucas H. M. Mens, Ad F. M. Snik, A. John van Opstal, Marc M. van Wanrooij

**Affiliations:** ^1^Department of Biophysics, Donders Institute for Brain, Cognition and Behavior, Radboud University, Nijmegen, Netherlands; ^2^Department of Otorhinolaryngology, Donders Institute for Brain, Cognition and Behavior, Radboud University Medical Center, Nijmegen, Netherlands

**Keywords:** bimodal, spatial hearing, speech-in-noise, electro-acoustic, residual hearing

## Abstract

This study describes sound localization and speech-recognition-in-noise abilities of a cochlear-implant user with electro-acoustic stimulation (EAS) in one ear, and a hearing aid in the contralateral ear. This listener had low-frequency, up to 250 Hz, residual hearing within the normal range in both ears. The objective was to determine how hearing devices affect spatial hearing for an individual with substantial unaided low-frequency residual hearing. Sound-localization performance was assessed for three sounds with different bandpass characteristics: low center frequency (100–400 Hz), mid center frequency (500–1,500 Hz) and high frequency broad-band (500–20,000 Hz) noise. Speech recognition was assessed with the Dutch Matrix sentence test presented in noise. Tests were performed while the listener used several on-off combinations of the devices. The listener localized low-center frequency sounds well in all hearing conditions, but mid-center frequency and high frequency broadband sounds were localized well almost exclusively in the completely unaided condition (mid-center frequency sounds were also localized well with the EAS device alone). Speech recognition was best in the fully aided condition with speech presented in the front and noise presented at either side. Furthermore, there was no significant improvement in speech recognition with all devices on, compared to when the listener used her cochlear implant only. Hearing aids and cochlear implant impair high frequency spatial hearing due to improper weighing of interaural time and level difference cues. The results reinforce the notion that hearing symmetry is important for sound localization. The symmetry is perturbed by the hearing devices for higher frequencies. Speech recognition depends mainly on hearing through the cochlear implant and is not significantly improved with the added information from hearing aids. A contralateral hearing aid provides benefit when the noise is spatially separated from the speech. However, this benefit is explained by the head shadow in that ear, rather than by an ability to spatially segregate noise from speech, as sound localization was perturbed with all devices in use.

## Introduction

Bimodal EAS listeners rely on a cochlear implant (CI) in one ear and bilateral acoustic amplification for spatial hearing in their everyday life. Normal-hearing listeners use interaural time (ITDs) and level (ILDs) differences to localize low frequency (< 1,500 Hz) and high frequency (> 3,000 Hz) sounds in the horizontal plane, respectively. Moreover, for broadband sounds, appropriate weighing of the ITDs and ILDs enables normal-hearing listeners to localize sounds accurately and precisely (1). However, the efficacy of hearing devices of bimodal EAS listeners to provide veridical ITD and ILD cues is not clear.

In this case study we present data collected from a bimodal EAS listener with rare and exceptionally-well, symmetric low-frequency residual hearing. Free-field horizontal sound-localization performance was tested with different device configurations for low-frequency (LF250 sound, bandwidth 100–400 Hz), mid-frequency (MF1000 sound: bandwidth 500–1500 Hz) and high-frequency broadband (HFBB sound; bandwidth 500–20,000 Hz) noise bursts. The residual low-frequency hearing (20 dB HL up to 250 Hz) of the listener potentially provides access to veridical ITDs, such that this listener, when unaided, might localize low-frequency sounds accurately. If, however, the ITDs are perturbed by acoustic amplification on either side, low-frequency sound-localization performance will deteriorate. For broadband sounds, sound localization is expected to depend on how the high- and low-frequency content will be weighted to form a spatial percept. Furthermore, as the higher frequencies will be audible mainly unilaterally via the CI while the lower frequencies will be audible bilaterally via the hearing aids, the devices are likely to introduce asymmetric hearing for the higher frequencies. Such asymmetry might lead to impoverished spatial hearing.

We also assessed speech recognition in noise in three spatial configurations with or without the use of hearing aids. Speech always came from the front, while noise was co-located with the speech or positioned on either the implanted or the hearing-aid side. Speech recognition was also measured in the CI-only condition, for speech and noise co-located straight ahead. We hypothesize that speech understanding depends predominantly on CI-use, and much less on acoustic amplification.

## Methods

### Participant

**Figure 1:**
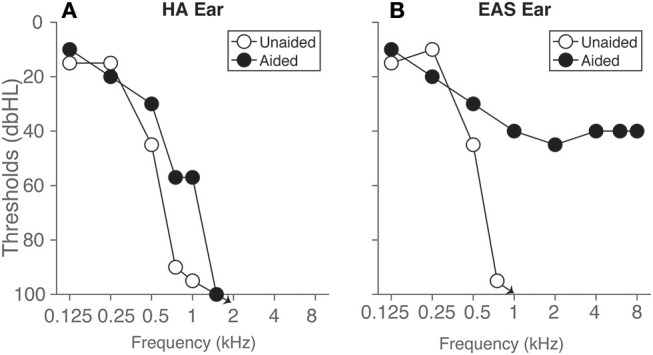
Hearing thresholds of the listener are presented for the ear **(A)** with hearing aid (HA ear) and the ear **(B)** with the EAS device. Open circles represent unaided post-operative pure-tone thresholds measured over headphones, and filled circles represent aided free-field warble tone thresholds, respectively. Arrows denote that the thresholds were 100 dB HL and above.

The listener was a female, 67 years, with a ski slope sensorineural hearing loss in both ears (Figure 1). In the right ear, she used an electro acoustic stimulation (referred to as “EA” in the following) device (Med-El Sonata) with a FLEXEAS (FLEX24) implant and a Duet 2 EAS sound processor. In the left ear, acoustic stimulation (abbreviated to “A” in the following) was provided by a Phonak Naida hearing aid (HA). She has been using her devices for over 5 years at the time of this study and her residual hearing has been consistent over time making it a unique case. Conventional earmolds were used in both ears with 2 mm venting. The aided and unaided audiograms of the listener are presented in Figure 1. Unaided thresholds (open circles) and aided thresholds are approximately symmetric across ears up to 500–1000 Hz. Aided thresholds for high frequencies (>1,000Hz) in the EA ear are around 40 dB HL. Electrical stimulation was provided with a low cut-off frequency of 639 Hz, and the acoustic amplification was provided up to 750 Hz. The contralateral hearing aid provided amplification up to 1,000 Hz.

### Sound Localization

#### Set-Up

Sound-localization experiments were carried out in a dark sound-treated room with a background noise level of <30 dB(A). In the room a spherical-shaped wire structure with a radius of 1.3 m contained 125 loudspeakers (2). The speakers were positioned on an orthogonal double-pole azimuth-elevation spherical grid (3) with 5 degrees spacing between the speakers along the cardinal axes, and 15 degrees spacing for rest of the speaker locations. Sounds with different spectra were presented in random order from randomly selected speaker locations, selected by custom-made MATLAB scripts. Sounds were delivered by TDT system 3 hardware (Tucker Davis Technologies, Alachua, FL, USA). Head movements were recorded with the search-coil technique to register the listener's sound-localization responses [e.g., (2)].

#### Stimulus Generation

Stimuli consisted of Gaussian white noise bursts with a duration of 150 ms. Noise bursts had 5 ms sine-squared onset and cosine-squared offset ramps. Sounds were bandpass filtered to create low-frequency band-pass sounds with a low center frequency of 250 Hz (LF250; bandwidth from 100 to 400 Hz), a mid-center frequency of 1,000 Hz (MF1000; 500–1500 Hz) and high-frequency broadband sounds (HFBB; 500-20,000 Hz).

#### Paradigm

Sounds were presented from 16 speaker locations ranging from −75 to +75 in the horizontal plane in 10 steps, at a level of 65 dBA as measured at the position of the listener's head with a calibrated sound-level meter (ISO-TECH SLM 1352 P). A green fixation LED was presented straight ahead before the start of each trial. The listener had to point a red laser pointer mounted on a head-fixed spectacle frame, toward the green fixation light. The listener operated a button box to start a new trial. Pressing the button extinguished the fixation light, and triggered a 150 ms duration noise burst after a 200 ms delay. The listener was instructed to orient the head toward the perceived direction of the sound as quickly and as accurately as possible. After each goal-directed head movement, the green fixation light was presented again to start the next trial. An acquisition time of 2 s was set to record the head movements.

We tested the following four listening conditions:

- Completely unaided, in which all devices were turned off (denoted by the symbol X), and the ear canals unoccluded.
- Fully aided, with acoustic (A) stimulation in the contralateral hearing aid ear and electro-acoustic (EA) stimulation in the CI ear.
- Electro-acoustic stimulation (EA) only with contralateral acoustic amplification (A) from the hearing aid ear taken off.
- Acoustic amplification (A) only with EA taken off.

#### Data Analysis

We analyzed the stimulus-response relations by fitting the data with the following linear relationship

(1) $$\alpha_{r}\;=\;b+\;g\alpha_{t}$$

where α_r_ and α_t_ are the azimuth response and stimulus locations, respectively (in degrees). The dimensionless slope, g, is referred to as the gain of the stimulus-response relationship; a gain of 1 is indicative for a perfect stimulus response relationship. The intercept, b, is the response bias in degrees, and will be zero in case of perfect localization. A positive (rightward) bias would mean that sounds tended to be localized toward the CI side, while a negative bias (leftward) would indicate perception toward the HA side. We also calculated the response variability (σ_*r*_) by taking the standard deviation of the residuals between the data and the optimal regression line. We used the root mean-squared error (RMSE in degrees) across all trials to quantify the overall accuracy of the responses.

### Speech Recognition

#### Paradigm

Speech recognition in noise was measured in the same recording session as the sound localization test. We used the Dutch Matrix sentence-in-noise test (4, 5) for two listening conditions. A test was performed with all devices on (A–EA), and a second test in the CI-only condition (X– EX). In the A–EA condition, three spatial configurations were measured. Speech was always presented from the front (0 deg), the noise was either co-located with the speech at 0 deg (referred to as S0N0) or presented at −90 deg (HA side, S0NHA) or +90 deg (EA side, S0NCI). For X–EX, we only tested the situation with noise and speech co-located at straight ahead, and compared it with the fully aided condition in order to assess the benefit of the acoustic amplification.

Three lists of 10 sentences were presented for each spatial configuration. Each sentence consisted of five words, randomly selected from a list, but arranged in a fixed grammatical order (for example a sentence translated in English: “Mark gives five large flowers”). The sentences were presented at a fixed level of 65 dB(A). The noise level was adapted according to the procedure described by (6) in to obtain a 50% correct word recognition per sentence. The signal-to-noise ratio (SNR) for each sentence was calculated by subtracting the noise level from the speech level [65 dB(A)]. The starting level of the noise was 60 dB(A) and the SNR was +5 dB(A). Prior to the start of the measurement, a practice run comprising 10 sentences was carried out, presented once in quiet, and once in the presence of background noise. The noise used was the International Female Fluctuating Masker.

#### Set-Up

The listener sat at a distance of 1.2 m from 3 loudspeakers (Tannoy, Reveal 402, London UK) located in front and at +/−90 on either side of the listener.

#### Analysis

Each of the three lists presented per condition tracked for 50 % correct word recognition. The last four responses of each list were taken to calculate the mean speech recognition threshold (SRT) and the 95% confidence intervals of the mean was also calculated.

Spatial release from masking (SRM) was calculated by taking the difference in speech recognition thresholds between S0N0 and S0NHA or S0NCI.

## Results

### Sound Localization

**Figure 2:**
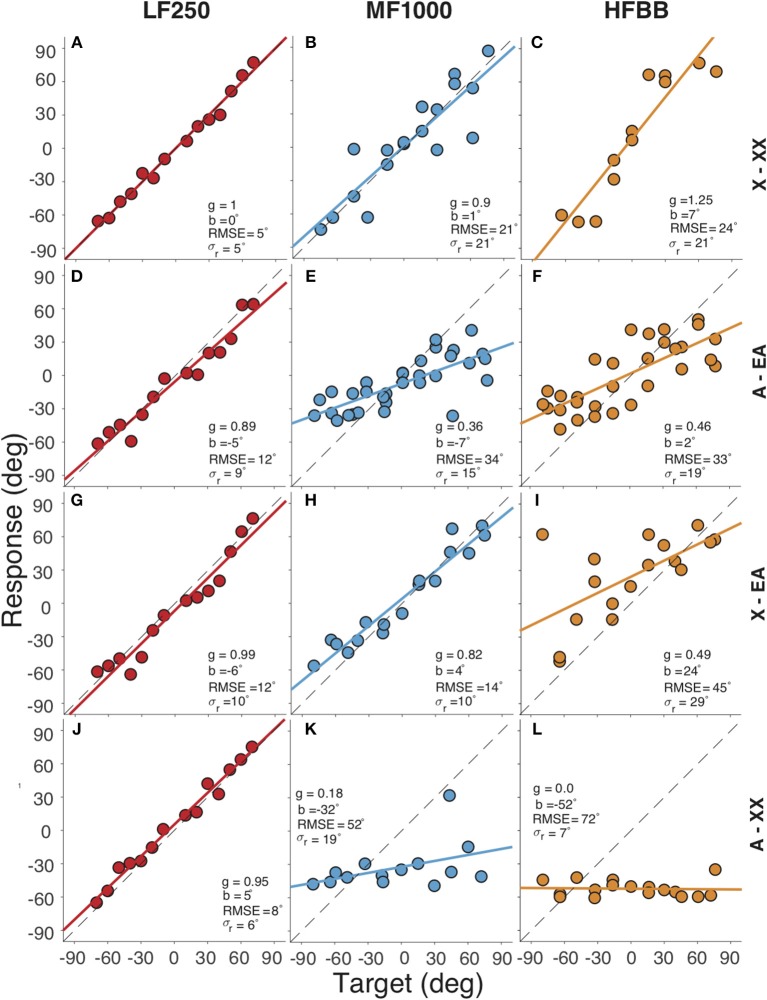
Target-response plots for four listening conditions (rows) and three sounds types (columns). Rows correspond to **(A–C)** completely unaided (X–XX) hearing, **(D–F)** fully aided (A–EA), **(G–I)** EA only (X–EA), **(J–L)** contralateral HA only (A–XX), respectively. Columns correspond to the **(A,D,G,J)** LF250, **(B,E,H,K)** MF1000 and **(C,F,I,L)** HFBB sounds. Colored circles indicate the localization response of the listener for each target location, lines denote the best-fit regression lines. In each plot, the RMSE and the regression parameters are listed: the bias (b), the gain (g), the response variability (**σ**_***r***_).

Figure 2 presents the target-response plots of the listener for the four different listening conditions. Sound localization for the completely unaided condition (denoted by X–XX in the figure) is presented in the top row. The listener localized the LF250 sounds with g = 1.00, b = 0°, RMSE = 5°, σ_*r*_ = 5 (Figure 2A), the MF1000 sounds with g = 0.90, b = 1°, RMSE = 21°, σ_*r*_ = 21 (Figure 2B) and the HFBB sounds with g = 1.25, b = 7 °, RMSE = 24°, σ_*r*_ = 21 (Figure 2C). Thus, the LF250 sounds were localized best with a very low mean absolute error compared to the MF1000 and HFBB sounds.

Localization for the fully-aided condition (denoted by A–EA) is shown in the second row. Again, the listener could accurately localize the LF250 sounds with all devices turned on (Figure 2D). When the stimulus had higher-frequency content, i.e., for the MF1000 (Figure 2E) and HFBB (Figure 2F) sounds, localization performance deteriorated (e.g., the gain dropped from g = 0.9 to g = 0.36 for MF1000, and from g = 1.25 to g = 0.46 for HFBB sounds, respectively). These results show that accurate and precise sound localization is achieved when all the devices are turned on for the very low-frequency sounds (LF250), but to a lesser extent for noises with high-frequency content (MF1000 and HFBB sounds). The introduction of the devices impoverished spatial hearing for the mid and high-frequency sounds (MF1000 and HFBB, cf. top and second row).

The third row of Figure 2 presents the sound localization for the unilaterally aided condition (denoted by X–EA), with only the EA active and the contralateral hearing aid removed. Again, the listener localized LF250 noise bursts accurately. Interestingly, also the MF1000 sounds were now accurately localized (g = 0.82, b = 4°, RMSE = 14°, σ_*r*_ = 10), while localization responses to HFBB noise were worse and biased toward the EA side (g = 0.48, b = 24°, RMSE = 45°, σ_*r*_ = 29). The improved localization for the mid-frequency noise bursts suggests that the contralateral hearing aid perturbs the sound localization cues available up to 1,500 Hz (cf. panel E vs. H).

The fourth row of Figure 2 presents the results of the unilaterally aided condition: the contralateral hearing-aid switched on, with the EA device turned off (denoted by A–XX). In this case, the location percept of the MF1000 and HFBB sounds was dominated by the contralateral hearing aid, as reflected by the large leftward biases of b = −32°and b = −52°, respectively, and the low response gains of g = 0.18 and g = 0.0, respectively.

### Speech Recognition

**Figure 3:**
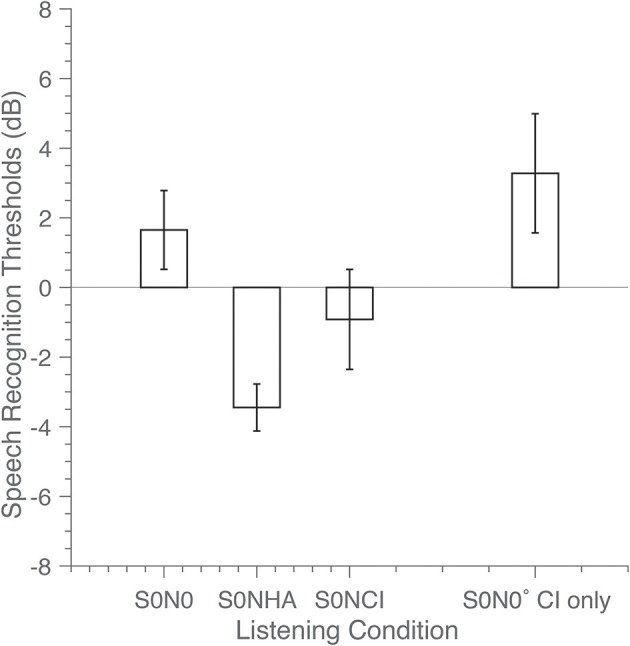
Speech reception thresholds (SRT) in dB were determined in the fully aided condition for three spatial configurations: speech and noise co-located straight ahead (S0N0), and for the noise located at + (S0NCI) or −90° (S0NHA). In the CI-only condition only one spatial configuration was tested (co-located, S0N0 CI only). Bars indicate SRT, with error bars representing the 95% confidence interval of the mean.

The speech-recognition thresholds are presented in Figure 3. For the A–EA listening condition, the SRT (with 95 % confidence interval) is 1.5 ± 1.3 dB (S0N0), −3.2 ± 0.6 dB (S0NHA) and −0.8 ± 1.4 dB (S0NCI). SRM is calculated as the difference in SRTs between the co-located and separated spatial configurations; the SRM is 4.7 dB when the noise is presented at the left, hearing-aid side, and 2.3 dB when the noise was presented at the right, EA side.

In the CI-only listening condition X – EX in the S0N0 configuration, the signal-to-noise ratio was 3.2 ± 1.5 dB. The SRT did not significantly improve (*p* = 0.09) for the fully aided A – EA condition with additional acoustic amplification, in comparison to CI-only hearing X - EX The measurement for the completely unaided condition (not shown) was discontinued because the listener could not recognize the sentences. This illustrates that the cochlear implant was required for speech recognition in noise for this listener.

## Discussion

The case presented here is unique because the listener had near-normal low-frequency residual hearing in both ears. Access to low-frequency spatial cues is the key feature of this case, which is discussed with respect to sound localization and speech recognition in noise in the following section.

### Access to ITDs

The observed near-normal sound localization to LF250 and MF1000 sounds in the unaided condition (Figure 2, first row), and to LF250 sounds for all listening conditions (Figure 2, first column) demonstrates that the listener has access to unperturbed, veridical ITDs at low frequencies. This is corroborated by the symmetric, near-normal low-frequency hearing thresholds up to 250 Hz (Figure 1). As high frequencies were inaudible for the listener in the unaided condition, the listener also used these ITDs effectively when localizing HFBB sounds.

In the fully aided condition, audibility of low frequencies between 250 and 750 Hz was enhanced through bilateral acoustic amplification, and audibility of high frequencies (exceeding 693 Hz) was enhanced through electrical stimulation on the EA side (Figure 1). The results indicate that device use negatively affected the listener's ability to localize sounds containing higher frequencies (MF1000 and HFBB; second and fourth rows of Figure 2). It is likely that the devices potentially introduced high-frequency sound-localization cues (ILDs), because of improved high-frequency audibility. However, these cues became unreliable for frequencies above 1,000 Hz, for lack of audibility on the HA side at those frequencies, yielding asymmetric hearing. The data suggest that these (perturbed) level cues are weighted across frequency bands, together with the ITD cues, to create an auditory spatial percept. Because of this integration, however, a less precise (larger RMSE, larger response variability) and less accurate (poor gain) localization response results for higher-frequency sounds as soon as any, or all, devices are turned on.

Dorman et al. (7) reported localization errors from a bilateral EA user that were the same with and without the hearing devices for low-pass (RMSE = 20°) and HFBB sounds (RMSE = 24°). Another study with 8 bimodal EA users (8) reported similar errors (RMSE of = 22°) for the fully aided condition for (very) low-pass sounds (125–500 Hz). In the present study, we report RMSE errors of just 5° in response to LF250 for unaided condition and 11° in the fully aided condition. Note that the listener in the present study had better and more symmetric residual hearing (15 dB HL up to 250 Hz for both ears), compared to the case discussed in Dorman et al. (7), who reported 45 dB HL (left ear) and 32.5 dB HL (right ear). It is likely that the better and more symmetric hearing thresholds were the underlying cause for better ITD perception and superior localization performance for the current case in the unaided condition.

#### Speech Recognition

The listener did not benefit from the acoustic devices in the speech-recognition task when speech and noise were co-located (Figure 3). This observation contrasts with the literature (9–11), which reports significant benefits of up to 2 dB between with or without acoustic amplification. Note again, that the listener in this report had near-normal low-frequency hearing, and even without acoustic amplification might have had access to the fundamental frequency of speech.

The listener had good access to localization cues in the unaided condition, which deteriorated with device use. In contrast, the benefit of the devices for the listener in recognizing speech was obvious. This finding suggests that the listener did not rely on ITDs for speech recognition in noise, and that the CI was the major, dominating source for the audibility of the speech signal.

The SRM of 4.7 dB compares well to the acoustic head-shadow effect for broad-band speech signals, as reported in the literature (12, 13). The SRM is less pronounced when the noise is presented at the EA ear, because the contralateral ear with hearing aid in the head shadow had access to low-frequency information only, and, as a result, speech recognition (and the SRT) was expected to be poor in that ear.

We conclude that the devices improved audibility of frequencies above 250 Hz, enabling significant improvement in speech recognition in noise. The audibility benefit also introduced ILDs, but these did not lead to a benefit in sound-localization performance. As suggested, these ILDs likely conflicted with the unperturbed ITDs, leading to an impaired sound-localization ability of the listener for all stimuli containing mid and high frequencies. Sound localization remained accurate and precise for low-frequency sounds, suggesting that acoustic and electrical amplification at higher frequencies, and the insertion of the CI electrode, did not affect ITD processing at frequencies where hearing was preserved.

The bimodal EAS listener with near-normal and symmetric low-frequency residual hearing presents a rare, but interesting case. This report emphasizes the importance of hearing preservation; without low-frequency residual hearing, adequate ITD processing would not be possible, and low-frequency sound localization would probably be impaired.

Remarkably, the subject reported that the devices (CI and HA) were used occasionally, only during conversations. When she was out in traffic, however, she preferred to use no device. These choices by the subject might be related to the reported outcomes of this study.

Given these findings, the possibility of implanting a second EAS device should be considered for similar listeners. Bilateral EAS devices potentially provide symmetric hearing even for the higher frequencies, which potentially allow for reliable ILDs across the (restored) hearing range, and might enable spatial hearing in the aided condition as well.

## Data Availability

All data are available from the Donders Institute for Brain, Cognition and Behavior repository at: http://hdl.handle.net/11633/aaci5jrc.

## Ethics Statement

All experimental procedures were approved by the local Ethics Committee of the Faculty of Social Sciences of the Radboud University (protocol nr. ECSW2016-2208-41). The listener gave her full written informed consent, prior to participation in the experiment. A written informed consent was also obtained from the participant for the publication of this case report.

## Author Contributions

LM, AS, AvO, and MvW contributed to the conception of the work. SS, LM, and MvW contributed to the experimental design. SS collected data. SS and MvW performed the analysis. All authors contributed to the interpretation of the data and were involved in writing the manuscript.

### Conflict of Interest Statement

The authors declare that the research was conducted in the absence of any commercial or financial relationships that could be construed as a potential conflict of interest.
